# A prognostic nomogram to predict survival in elderly patients with small-cell lung cancer: a large population-based cohort study and external validation

**DOI:** 10.1186/s12885-022-10333-9

**Published:** 2022-12-06

**Authors:** Guangrong Lu, Jiajia Li, Yejiao Ruan, Yuning Shi, Xuchao Zhang, Yushan Xia, Zheng Zhu, Jiafeng Lin, Lili Li

**Affiliations:** 1 Department of Gastroenterology, The Second Affiliated Hospital and Yuying Children’s Hospital of Wenzhou Medical University, Wenzhou, China; 2 The Second Clinical Medical College of Wenzhou Medical University, Wenzhou, China; 3 Cardiovascular Department, The Second Affiliated Hospital and Yuying Children’s Hospital of Wenzhou Medical University, Wenzhou, 325000 China; 4 Departments of Medical Oncology, The First Affiliated Hospital of Wenzhou Medical University, No.2 Fuxue Lane, Wenzhou, 325000 China

**Keywords:** Small cell lung cancer, Nomogram, Survival prediction, Elderly patients, Cancer staging

## Abstract

**Background:**

Age is an independent prognostic factor for small cell lung cancer (SCLC). We aimed to construct a nomogram survival prediction for elderly SCLC patients based on the Surveillance, Epidemiology, and End Results (SEER) database.

**Methods:**

A total of 2851 elderly SCLC patients from the SEER database were selected as a primary cohort, which were randomly divided into a training cohort and an internal validation cohort. Additionally, 512 patients from two institutions in China were identified as an external validation cohort. We used univariate and multivariate to determine the independent prognostic factors and establish a nomogram to predict survival. The value of the nomogram was evaluated by calibration plots, concordance index (C-index) and decision curve analysis (DCA).

**Results:**

Ten independent prognostic factors were determined and integrated into the nomogram. Calibration plots showed an ideal agreement between the nomogram predicted and actual observed probability of survival. The C-indexes of the training and validation groups for cancer-specific survival (CSS) (0.757 and 0.756, respectively) based on the nomogram were higher than those of the TNM staging system (0.631 and 0.638, respectively). Improved AUC value and DCA were also obtained in comparison with the TNM model. The risk stratification system can significantly distinguish individuals with different survival risks.

**Conclusion:**

We constructed and externally validated a nomogram to predict survival for elderly patients with SCLC. Our novel nomogram outperforms the traditional TNM staging system and provides more accurate prediction for the prognosis of elderly SCLC patients.

**Supplementary Information:**

The online version contains supplementary material available at 10.1186/s12885-022-10333-9.

## Background

Worldwide, lung cancer remains a major public health problem and the leading cause of cancer-related deaths [1]. There are generally two main types of lung cancer; Non-small cell lung cancer (NSCLC) accounts for 85% of cases and small cell lung cancer (SCLC) for 15% [2]. SCLC is a highly aggressive neuroendocrine carcinoma with rapid doubling time, early metastasis and poor prognosis. Despite initial sensitivity to chemotherapy, most patients tend to develop treatment resistance quickly, followed by relapse and eventual death [3]. In recent years, immune checkpoint inhibitors (ICIs) have changed the treatment modality for SCLC to improve overall survival. However, this immunochemotherapy strategy does not benefit as well in SCLC as in metastatic NSCLC, possibly due to the immunosuppressive phenotype of SCLC [4].

Age is an independent prognostic factor for SCLC [5–7]. Previous studies have shown that compared with younger patients, elderly patients are less tolerant to surgery, chemotherapy and radiotherapy, and thus have poorer compliance with anti-tumor therapy and increased side effects [6, 8]. Organ aging accompanied by decreased immune function in elderly patients may be responsible for the increased risk of tumor recurrence [9, 10]. In clinical practice, patients aged ≥60 years often receive more conservative treatment. Current evidence guiding the management of elderly SCLC patients refers to data from all patients and may not be applicable to some patients. The American Joint Committee on Cancer (AJCC) TNM (Tumor-Node-Metastasis) staging system is a common tool used by oncologists for predicting tumor progression and develop treatment plans [11]. However, it has several drawbacks, as various factors such as gender, age, location, and treatment modality can affect individual survival outcomes for cancer patients [8, 12–16]. Therefore, it is needful to construct a comprehensive prognostic model including the AJCC staging system to better predict the prognosis of patients.

Nomogram is considered a reliable tool to visually assess the risks by integrating important pathological and clinical features of oncologic outcomes [17, 18]. Additionally, compared with the traditional AJCC staging system, nomograms have been shown to provide more precise predictions for several cancers [19–22]. However，few nomograms have been used to predict survival outcomes in elderly SCLC. A recent study has established a nomogram for predicting survival of SCLC patients aged ≥65 years [8]. However, the study only included stage I SCLC patients, and the nomogram had not undergone external validation. The aim of our study was to develop a new nomogram to quantify the prognosis of elderly SCLC using a cohort from population-based Surveillance, Epidemiology, and End Results (SEER) program, and externally validate it with an independent cohort of patients.

## Methods

### Patient population

The flow chart of this study was shown in Additional file 1. The SEER database (https://seer.cancer.gov/) includes 18 population-based cancer registries covering 28% of the US population. Data between 1975 and 2017 were collected using SEER*Stat software (v 8.3.5). Patients with SCLC and at least 60 years of age at diagnosis were included in this analysis. The following variables were assessed: age, sex, race, marital status, insurance, tumor location, histology grade, the 7th TNM stage (published in 2010), surgery, chemotherapy, radiation, metastatic sites, tumor size, follow-up time, cancer-specific death, and all-cause death. We excluded patients with incomplete information on the above variables. Overall survival (OS) and cancer-specific survival (CSS) were defined as the time from diagnosis to the last follow-up or death from all causes or cancer-related death, respectively.

An external validation cohort was constructed to test the generality of the prognostic model. The cohort included 512 eligible cases diagnosed at two institutions (the First Affiliated Hospital of Wenzhou Medical University and the Second Affiliated Hospital of Wenzhou Medical University) in Wenzhou from 2007 to 2017. Independent prognostic variables according the training cohort were collected. This study was approved by the Ethics Committees of the First Affiliated Hospital of Wenzhou Medical University and the Second Affiliated Hospital of Wenzhou Medical University. As this study was designed retrospectively, informed consent was not required.

### Nomogram construction and statistical analyses

We used Pearson’s χ2 or Fisher’s Exact test to determine differences in baseline characteristics between the training cohort and the internal validation cohort. We performed univariate and multivariate cox proportional hazard regression analyses to identify the variables affecting CSS and OS in the training group. We utilized the prognostic factors determined in the multivariate analysis to construct the nomogram and then test its ability to predict 1-, and 2-year survival in SCLC patients by internal and external validation cohorts, respectively.

We used the concordance index (C-index) and area under the receiver operator characteristic (ROC) curve (AUC) to determine the discrimination of the nomogram. Calibration curves were drawn to evaluate the consistency between actual outcome and predicted survival. We used decision curve analysis (DCA) to compare the advantages and improved performance of the nomogram and the AJCC staging system. Patients were divided into high risk and low risk groups based on the nomogram risk score. The cut-off point of risk stratification was obtained from the ROC curve analysis. Kaplan-Meier survival ananlysis with the log-rank test was utilized to evaluate the significance of survival difference between the high and low risk groups. All statistical analyses were conducted on R version 3.4.2 software. All tests were two-sided, and *P* < 0.05 was considered statistically significant.

## Results

### Patient characteristics

We enrolled 2851 eligible SCLC patients aged over 60 for this study. The main cohort was randomly divided into two groups in a 7:3 ratio, training cohort (*N* = 1999) and an internal validation cohort (*N* = 852). In the training cohort, the majority of patients were 60–69 years old (45.6%), female (52.5%), white (87.6%), married (51.8%), and insured (86.1%). The main tumor site was the upper lobe (56.6%) of the lung. The tumors were mostly at histologic grades III and IV (*n* = 1195, 59.8%), while stage IV (*n* = 1207, 60.4%) was the most common AJCC stage. The proportions of patients who received surgery, chemotherapy, and radiotherapy were 6.9, 71.4 and 48.7%, respectively. There were 399(20.0%), 295(14.8%), 488(24.4%)and 263(13.2%) patients with bone, brain, liver and lung metastasis, respectively. Patients were comparable between the training set and internal validation set for all clinicopathological features (Table 1).

**Table 1:** Patient characteristics

Variables	Total (***n*** = 2851)	Training Cohort (***n*** = 1999)	Validation Cohort (***n*** = 852)	***P value***
Age (year)n(%)				0.521
60–69	1297(45.5)	912(45.6)	385(45.2)	
70–79	1145(40.2)	792(39.6)	353(41.4)	
≥ 80	409(14.3)	295(14.8)	114(13.4)	
Sex n(%)				0.472
Female	1485(52.1)	1050(52.5)	435(51.1)	
Male	1366(47.9)	949(47.5)	417(48.9)	
Race n(%)				0.993
Black	242(8.5)	170(8.5)	72(8.5)	
White	2497(87.6)	1751(87.6)	746(88.7)	
Others	112(3.9)	78(3.9)	34(3.8)	
Marital status n(%)				0.838
Married	1474(51.7)	1036(51.8)	438(51.4)	
Unmarried/single	1377(48.3)	963(48.2)	414(48.6)	
Insurance n(%)				0.368
Insured	2442(85.6)	1722(86.1)	720(84.5)	
Any medicaid	361(12.7)	242(12.1)	119(14.0)	
Uninsured	48(1.7)	35(1.8)	13(1.5)	
Location n(%)				0.120
main bronchus	301(10.6)	195(9.8)	106(12.4)	
upper lobe,lung	1582(55.5)	1132(56.6)	450(52.8)	
middle lobe,lung	139(4.9)	102(5.1)	37(4.3)	
low lobe,lung	776(27.2)	532(26.6)	244(28.6)	
overlapping lesion	53(1.8)	38(1.9)	15(1.9)	
Grade n(%)				0.122
I	43(1.5)	26(1.3)	17(2.0)	
II	1082(38.0)	778(38.9)	304(35.7)	
III/IV	1726(60.5)	1195(59.8)	531(62.3)	
AJCC TNM stage(7th) n(%)				0.326
I	190(6.7)	135(6.7)	55(6.5)	
II	147(5.1)	96(4.8)	51(5.9)	
III	778(27.3)	561(28.1)	217(25.5)	
IV	1736(60.9)	1207(60.4)	529(62.1)	
Surgery n(%)				0.698
No	2659(93.3)	1862(93.1)	797(93.5)	
Yes	192(6.7)	137(6.9)	55(6.5)	
Chemotherapy n(%)				0.452
No/unknown	804(28.2)	572(28.6)	232(27.2)	
Yes	2047(71.8)	1427(71.4)	620(72.8)	
Radiation n(%)				0.426
No/unknown	1448(50.8)	1025(51.3)	423(49.6)	
Yes	1403(49.2)	974(48.7)	429(50.4)	
Bone metastasis n(%)				0.436
No	2271(79.7)	1600(80.0)	671(78.8)	
Yes	580(20.3)	399(20.0)	181(21.2)	
Brain metastasis n(%)				0.672
No	2425(85.1)	1704(85.2)	721(84.6)	
Yes	426(14.9)	295(14.8)	131(15.4)	
Liver metastasis n(%)				0.789
No	2151(75.4)	1511(75.6)	640(75.1)	
Yes	700(24.6)	488(24.4)	212(24.9)	
Lung metastasis n(%)				0.806
No	2473(86.7)	1736(86.8)	737(86.5)	
Yes	378(13.3)	263(13.2)	115(13.5)	
Size n(%)				0.315
≤ 3 cm	811(28.4)	576(28.8)	235(27.6)	
3.1-5 cm	789(27.7)	568(28.4)	221(25.9)	
5.1-7 cm	567(19.9)	389(19.5)	178(20.9)	
>7 cm	684(24.0)	466(23.3)	218(25.6)	

*AJCC* American Joint Committee for Cancer, *TNM* Tumor-Node-Metastasis

In the external validation cohort, 245(47.9%) patients were aged 60–69 years, and 237(46.3%) patients were male. Among these patients, 52 (10.1%), 409 (79.9%) and 287 (56.0%) received surgery, chemotherapy, and radiotherapy, respectively. The majority of patients were stage IV with distant metastasis (Supplementary Table 1).

### Independent prognostic factors

Table 2 showed the results of univariate and multivariate analyses. All significant factors of OS and CSS in the univariate analysis were accessed into the multivariate analysis. The multivariate analysis showed that sex (*P* = 0.023), age (*P* < 0.001), AJCC stage (*P* < 0.001), surgery (*P* < 0.001), chemotherapy (*P* < 0.001), radiation (*P* < 0.001), bone metastasis (*P* = 0.037), brain metastasis (*P* < 0.001), liver metastasis (*P* < 0.001) and tumor size (*P* < 0.001) were independent prognostic factors for both OS and CSS. Other variables identified in the univariate analysis, such as insurance, marital status, tumor location and grade, were not independent factors for either OS or CSS.

**Table 2:** Univariate and multivariate analysis for survival in the training cohort

Variables	Overall survival				Cancer-specific survival			
	Univariate analysis		Multivariate analysis		Univariate analysis		Multivariate analysis	
	log rank X^**2**^	***P*** value	HR(95% CI)	***P*** value	log rank X^**2**^	***P*** value	HR(95% CI)	***P*** value
Sex	7.573	0.006			5.096	0.024		
Female			Reference				Reference	
Male			1.122(1.0160–1.239)	0.023			1.115(1.009–1.232)	0.033
Age (year)	60.828	< 0.001		< 0.001	57.346	< 0.001		< 0.001
60–69			Reference				Reference	
70–79			1.125(1.014–1.249)	0.027			1.105(0.991–1.232)	0.072
≥ 80			1.403(1.215–1.621)	< 0.001			1.422(1.225–1.651)	< 0.001
Race	3.169	0.205			2.239	0.326		
Black								
White								
Others								
Marital status	3.877	0.049			3.243	0.072		
Married			Reference					
Unmarried/single			0.943(0.852–1.044)	0.260				
Insurance	7.334	0.026		0.139	6.300	0.043		0.201
Insured			Reference				Reference	
Any medicaid			1.156(0.998–1.339)	0.053			1.136(0.977–1.322)	0.098
Uninsured			1.114(0.776–1.600)	0.558			1.158(0.802–1.673)	0.435
Location	13.801	0.008		0.890	13.333	0.010		0.912
main bronchus			Reference				Reference	
upper lobe, lung			0.981(0.834–1.155)	0.821			0.989(0.835–1.172)	0.900
middle lobe, lung			0.938(0.723–1.216)	0.627			0.909(0.691–1.195)	0.493
low lobe, lung			1.029(0.862–1.228)	0.753			1.021(0.849–1.227)	0.829
overlapping lesion			0.942(0.650–1.365)	0.752			0.968(0.661–1.418)	0.869
Grade	8.509	0.014		0.103	6.444	0.040		0.207
I			Reference				Reference	
II			1.217(0.935–1.584)	0.072			1.420(0.871–2.313)	0.160
III/IV			1.672(1.276–2.191)	0.046			1.487(0.915–2.417)	0.109
AJCC TNM stage(7th)	325.842	< 0.001		< 0.001	333.677	< 0.001		< 0.001
I			Reference				Reference	
II			1.057(0.759–1.472)	0.743			1.196(0.835–1.714)	0.328
III			2.020(1.583–2.579)	< 0.001			2.257(1.723–2.957)	< 0.001
IV			2.456(1.904–3.167)	< 0.001			2.816(2.128–3.726)	< 0.001
Surgery	94.156	< 0.001			93.865	< 0.001		
No			Reference				Reference	
Yes			0.455(0.356–0.580)	< 0.001			0.449(0.345–0.585)	< 0.001
Chemotherapy	399.497	< 0.001			357.865	< 0.001		
No/unknown			Reference				Reference	
Yes			0.358(0.318–0.403)	< 0.001			0.355(0.314–0.401)	< 0.001
Radiaton	228.240	< 0.001			200.382	< 0.001		
No/unknown			Reference				Reference	
Yes			0.646(0.578–0.723)	< 0.001			0.663(0.590–0.746)	< 0.001
Bone metastasis	108.425	< 0.001			117.648	< 0.001		
No			Reference				Reference	
Yes			1.145(1.008–1.301)	0.037			1.168(1.025–1.332)	0.020
Brain metastasis	37.292	< 0.001			38.774	< 0.001		
No			Reference				Reference	
Yes			1.339(1.159–1.547)	< 0.001			1.345(1.158–1.562)	< 0.001
Liver metastasis	237.796	< 0.001			244.918	< 0.001		
No			Reference				Reference	
Yes			1.490(1.316–1.689)	< 0.001			1.531(1.346–1.742)	< 0.001
Lung metastasis	52.925	< 0.001			54.613	< 0.001		
No			Reference				Reference	
Yes			1.128(0.979–1.300)	0.096			1.132(0.978–1.311)	0.097
size	78.492	< 0.001		< 0.001	85.026	< 0.001		< 0.001
≤ 3 cm			Reference				Reference	
3.1-5 cm			1.249(1.097–1.422)	0.001			1.285(1.121–1.473)	< 0.001
5.1-7 cm			1.297(1.121–1.501)	< 0.001			1.309(1.123–1.526)	0.001
>7 cm			1.365(1.185–1.572)	< 0.001			1.410(1.217–1.634)	< 0.001

*AJCC* American Joint Committee for Cancer, *TNM* Tumor-Node-Metastasis

### Nomogram construction

**Fig. 1:**
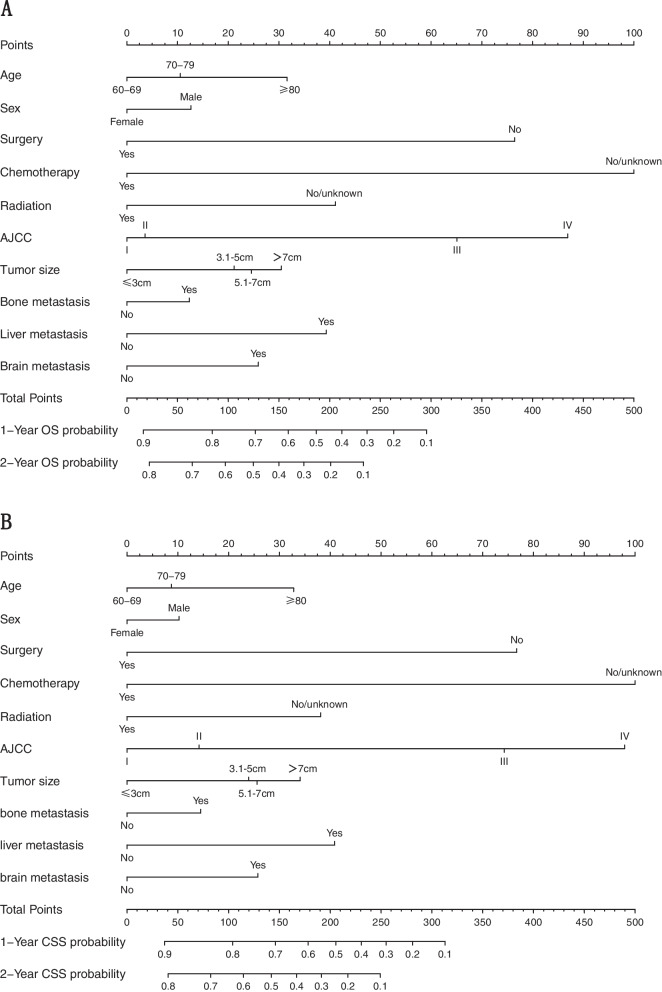
Nomogram predicting 1-, and 2-year OS (**A**) and CSS (**B**) of patients with small cell lung cancer

Ten prognostic indicators determined by multivariate analyses were used to develop the nomograms. Figure 1 demonstrated the nomograms for predicting the probability of the 1- and 2-year OS and CSS rates in the training cohort. The results indicated that chemotherapy was the strongest prognostic factor followed by AJCC stage and surgery. Each level of each predictor is scored on the nomogram. The total scores were calculated by adding the scores for each predictor, estimating the 1- and 2-year survival for individual patients on the basis of a vertical line from the total-points row.

### Calibration and internal validation

**Fig. 2:**
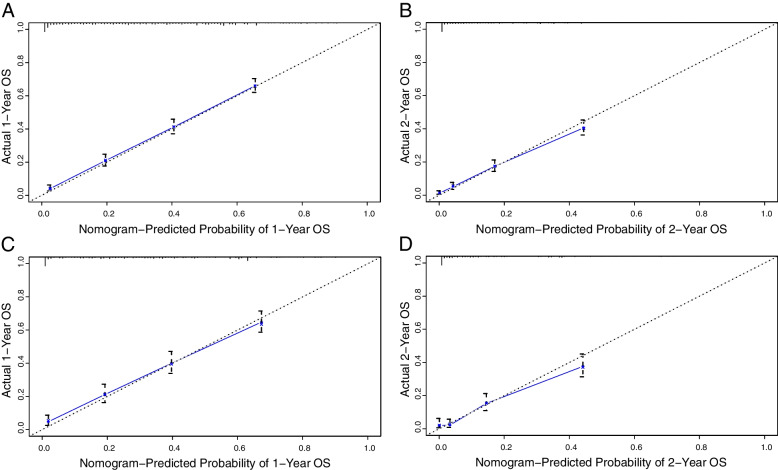
Calibration plots of the nomogram for 1-, and 2-year OS prediction of the training cohort (**A–B**) and internal validation cohort (**C–D**)

Calibration plots of 1- and 2-year OS probabilities in both the training and internal validation cohorts showed good consistency between the nomogram predicted survival and actual observations (Fig. 2). Similar results for CSS were shown in Supplementary Fig. 1. For the training cohort, the C-index of the established OS nomogram [0.751; 95% confidence interval (CI), 0.739–0.763] was better than the 7th TNM staging system (0.625; 95% CI, 0.611–0.639; *P* < 0.001). For the internal validation cohort, the C-index of the new nomogram (0.745; 95% CI, 0.725–0.765) also outperformed the traditional TNM model (0.622; 95% CI, 0.602–0.642; *P* < 0.001) (Table 3). A similar trend was also observed in CSS nomogram (Table 3).

**Table 3:** C-indexes for the nomograms and TNM stage system in elderly patients with SCLC

Survival		Training cohort	***P*** value	Internal validation cohort	***P*** value
Overall survival	Nomogram	0.751(0.739–0.763)	<0.001	0.745(0.725–0.765)	<0.001
	7th TNM stage	0.625(0.611–0.639)		0.622(0.602–0.642)	
Cancer-specific survival	Nomogram	0.757(0.745–0.769)	<0.001	0.756(0.736–0.776)	<0.001
	7th TNM stage	0.631(0.617–0.645)		0.638(0.618–0.658)	

*TNM* Tumor-Node-Metastasis, *C-index* Concordance index, *SCLC* Small cell lung cancer

### Comparison of the Nomogram and 7th TNM staging system

**Fig. 3:**
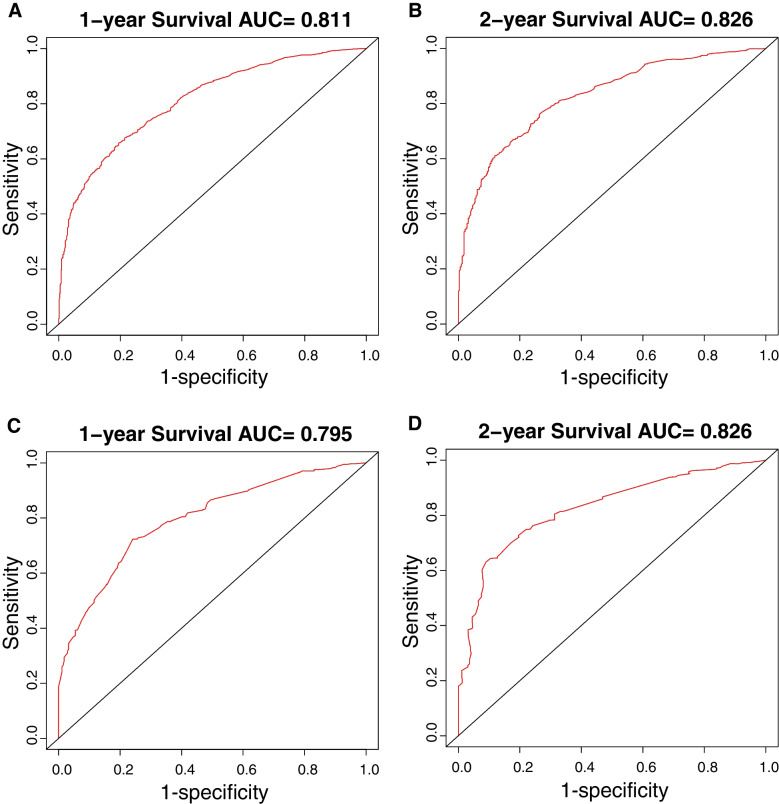
The ROC curves of the nomograms for 1-, and 2-year OS prediction of the training cohort (**A–B**) and internal validation cohort (**C–D**)

**Fig. 4:**
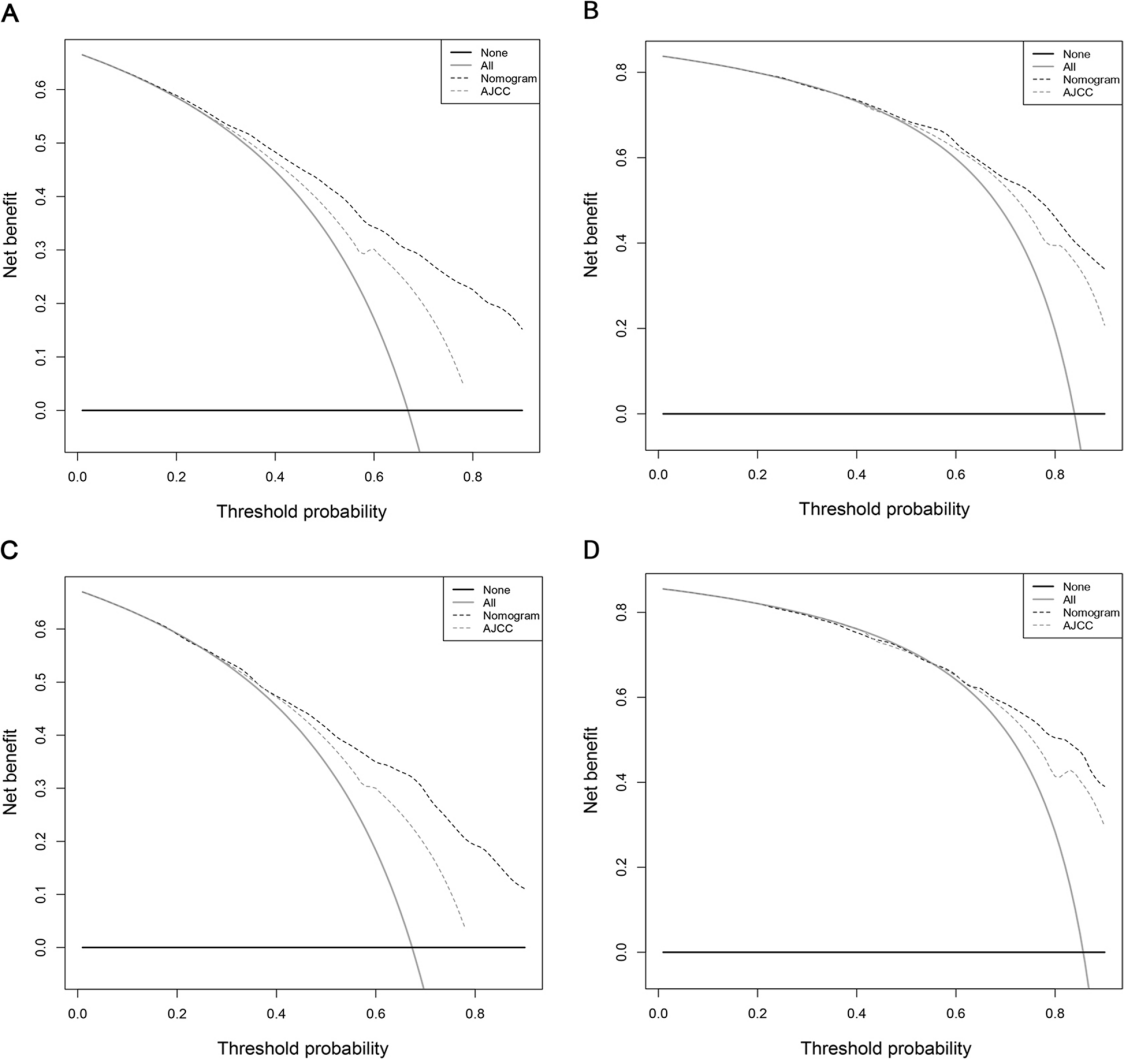
Decision curve analyses (DCA) of the nomogram and 7th AJCC TNM staging system for 1-year (**A, C**) and 2-year (**B, D**) overall survival in the training cohort (**A–B**) and internal validation cohort (**C–D**)

The AUC values of the 1- and 2-year OS nomogram is higher than that of the TNM staging both in the training group (1-year: 0.811 vs. 0.694, 2-year: 0.826 vs. 0.744) and internal validation group (1-year: 0.795 vs. 0.686, 2-year: 0.826 vs. 0.754) (Fig. 3 & Table 4). The related results for CSS were listed in Supplementary Fig. 2 and Table 4. The DCAs of OS and CSS compared the net benefits of the novel nomograms and the TNM staging system. Figure 4 and Supplementary Fig. 3 showed that 1- and 2-year outcomes of our nomograms outperformed those of the 7th TNM staging system in terms of various risk factors for death both in the training and internal validation groups. This indicated that our new model had better clinical utility and practical decision-making effects.

**Table 4:** Comparison of AUC values between nomograms and TNM stage system in elderly patients with SCLC

Survival		Training cohort		Internal validation cohort	
		1-year survival	2-year survival	1-year survival	2-year survival
Overall survival	Nomogram	0.811	0.826	0.795	0.826
	7th TNM stage	0.694	0.744	0.686	0.754
Cancer-specific survival	Nomogram	0.815	0.826	0.808	0.834
	7th TNM stage	0.699	0.745	0.701	0.762

*TNM* Tumor-Node-Metastasis, *SCLC* Small cell lung cancer

### External validation of nomogram

**Fig. 5:**
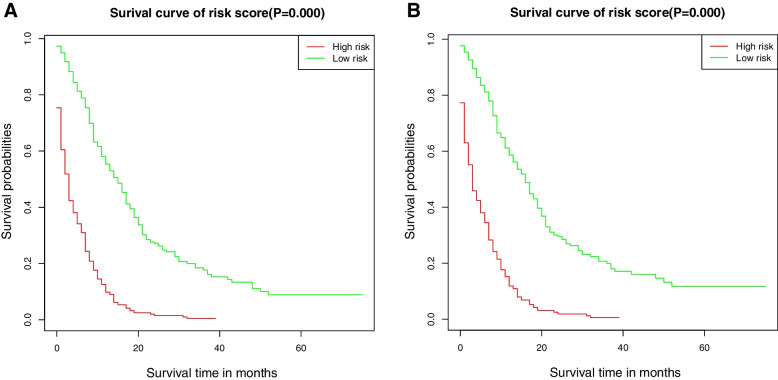
Kaplan–Meier curves of OS (**A**) and CSS (**B**) to test the risk stratification system in the external validation cohort

The nomograms were further externally validated in elderly SCLC patients diagnosed between 2007 and 2017 in two institutions. The C-index for OS and CSS prediction were 0.767 (95% CI, 0.745–0.789) and 0.769 (95% CI, 0.745–0.793), respectively. Our models demonstrated a good level of discriminative ability to predict 1- and 2-year OS (0.828 and 0.853) and CSS (0.836 and 0.854) (Supplementary Fig. 4). The calibration curves demonstrated an optimal agreement between predicted and actual observed probability of survival (Supplementary Fig. 5). Additionally, with the help of nomogram, patients were grouped into different risk stratification to evaluate the survival. The high-risk cohort had significantly worse OS and CSS than the low-risk cohort (*P* < 0.001) (Fig. 5).

## Discussion

SCLC is a subtype of lung cancer with poor prognosis, mainly occurring in elderly patients. Studies have shown that aging predicts a worse outcome, with elderly patients likely to have poorer performance status and more treatment-related toxicities [6, 8]. Therefore，age should be considered as an important variable in selecting therapeutic methods. However, current TNM staging criterion used to predict prognosis of SCLC patients ignore significant risk factors that can improve individualized survival predictions, such as age, gender, histologic grade, and treatment-related factors. In this study, we developed a more accurate nomogram based on a combination of independent prognostic factors to predict the probability of survival in elderly SCLC patients. Our novel nomogram incorporated ten variables: sex, age, AJCC stage, surgery, chemotherapy, radiation, bone metastasis, brain metastasis, liver metastasis and tumor size, which was able to provide more accurate assessment and prediction of elderly SCLC patients compared with the TNM staging criteria.

In this study, most elderly SCLC patients were white people aged 60–69 years. The tumor was mainly located in the upper lobe of the lung. Most patients had advanced AJCC staging with histological grade III and IV. A large proportion of elderly patients received chemotherapy, but less than 7% of cases underwent surgery. These were some unique disease features for elderly SCLC patients. In addition, we identified ten independent prognostic factors for OS and CSS, which were consistent with the previous findings [7, 13–16, 23–25]. Our model indicated that chemotherapy made the greatest contribution to the prognostic score. Multiple studies have proved that chemotherapy, as the most important treatment for SCLC, can prolong the survival time of patients [7, 13, 15, 23, 26]. Our analysis also indicated that AJCC stage and surgery had greater impacts on patient survival. Wang et al. established a prognostic nomogram for SCLC patients, in which AJCC stage made the greatest contributions to the final risk score [16]. A recent review also indicated that surgery has the greatest impact on the prognosis of patients with SCLC and should be recommended for certain patients, especially in the early stages [24].

Multiple studies have shown that risk factors such as increased age, male sex and larger tumor are negatively correlated with long-term survival [7, 13–15, 23, 25]. Zhong et al. constructed a novel predictive nomogram for extensive-stage SCLC patients by screening out independent prognostic factors such as gender, age, TNM staging and treatment methods [15]. Another study conducted by shan et al. identified seven prognostic factors and developed a predictive model for SCLC patients with brain metastasis [25]. These results were consistent with our study. In addition, the survival time of SCLC patients varies depending on the number and site of metastasis [27]. Nakazawa et al. found that extensive-stage SCLC most often metastasized to the liver, lung, brain, bone and adrenal gland [28]. They also demonstrated that patients with liver and multiple organ metastases had the worst survival outcomes. In accordance with these findings, our study showed that liver, bone and brain metastasis had a significant impact on the prognosis of elderly SCLC patients.

Validation of predictive models is critical to determine generalization and avoid overfitting [29]. In the current study, calibration plots showed a good agreement between the nomogram predicted and actual observed 1- and 2-year OS and CSS, which verified the repeatability and reliability of the established nomograms [21, 30]. The higher C-indexes and AUC values of the nomogram compared with the AJCC staging system indicated better discrimination ability of the nomograms. Besides, the C-index and AUC value in the validation cohort of our model were also higher than those of the previously SCLC nomogram published by Wang et al. (C-index: 0.745 vs. 0.722, AUC value: 0.826 vs. 0.789) [16]. Further DCA analyses also testified its obvious clinical application benefit versus traditional AJCC staging model. In addition, according to risk stratification model of this nomogram, patients in the external validation cohort can be effectively divided into high risk and low risk groups with distinguished OS and CSS. To our knowledge, this is the first nomogram survival prediction using SEER database and external validation cohort to predict survival in elderly SCLC patients. It also can be inferred from our study that the characteristics of a high-risk SCLC patient are: elderly male, late stage, large tumor, no surgery or radiotherapy or chemotherapy, with bone, liver or brain metastases. More importantly, our nomogram shows better ability and value than the TNM staging system. We believe that a well-designed nomogram can accurately predict each patient’s prognosis, thus benefiting both clinicians and patients.

Our study has some limitations. First of all, the data in this study was collected retrospectively, which may lead to unavoidable bias. Second, we did not include several treatment-related factors, such as chemotherapy regimens, numbers of cycles, doses and methods of radiation and targeted therapy, which could also influence the prognosis. Third, the external validation cohorts were all from the Asian population, and the sample size was relatively small. Future prospective clinical trials with larger sample size and different ethnic populations are necessary to validate our findings. Finally, it would be interesting to validate the already existing models from Wang et al. [16] on the SEER and our external datasets. However, the SEER database did not contain Charlson/Deyo score information, which prevented direct comparison of performance between our model and the published nomogram from Wang et al. Besides, we do not have the access to the NCBD database. Therefore, we compared the C-index and AUC value of our model with Wang’s nomogram and found that our model performed better. In spite of these shortcomings, our nomogram is established based on large population data collection from the SEER database, which provides a good opportunity to predict OS and CSS for elderly SCLC patients.

## Conclusions

In conclusion, we constructed and externally validated a nomogram to predict 1- and 2-year survival for elderly patients with SCLC. This novel nomogram outperforms the traditional TNM staging system and provides more accurate prediction for the prognosis of elderly SCLC patients.

## Supplementary Information

**Additional file 1.** The flow chart of this study.

**Additional file 2: Supplementary Fig. 1.** Calibration plots of the nomogram for 1-, and 2-year CSS prediction of the training cohort (A–B) and internal validation cohort (C–D).

**Additional file 3: Supplementary Fig. 2.** The ROC curves of the nomograms for 1-, and 2-year CSS prediction of the training cohort (A–B) and internal validation cohort (C–D).

**Additional file 4: Supplementary Fig. 3.** Decision curve analyses (DCA) of the nomogram and 7th AJCC TNM staging system for 1-year (A, C) and 2-year (B, D) CSS in the training cohort (A–B) and internal validation cohort (C–D).

**Additional file 5: Supplementary Fig. 4.** The ROC curves of the nomograms for 1-, and 2-year OS (A–B) and CSS (C–D) prediction in external validation cohort.

**Additional file 6: Supplementary Fig. 5.** Calibration plots of the nomogram for 1-, and 2-year OS (A–B) and CSS (C–D) prediction in external validation cohort.

**Additional file 7: Supplementary Table 1.** Patient characteristics of external validation cohort.

## Data Availability

The datasets generated for this study are available on request to the corresponding author.
