# Supplementary material for: A prognostic nomogram to predict survival in elderly patients with small-cell lung cancer: a large population-based cohort study and external validation
Source: BMC Cancer. 2022 Dec 6;22:1271. doi: 10.1186/s12885-022-10333-9 (PMC9724365; doi:10.1186/s12885-022-10333-9)
Supplement: Supplementary file 1 — Additional file 1. The flow chart of this study. [file 12885_2022_10333_MOESM1_ESM.doc]

**The flow chart of this study**

Total cases of elderly patients with small cell lung cancer from SEER between 1975 and 2017(n=63837)

Exclude patients with incomplete AJCC 7^th^ TNM stage (n=26739)

Exclude patients with multiple primaries tumors(n=19985)

Exclude patients with incomplete survival data, missing data in SEER cause-specific death classification, unknown grade, unknown surgery, unknown race, unknown marital status, unknown insurance,unknown remote metastasis(n=2851)

Training set

(n=1999)

Validation set

(n=852)

Total cases of elderly patients with small cell lung cancer from two institutions between 2007 and 2017(n=821)

Exclude patients with incomplete AJCC 7^th^ TNM stage (n=767)

Exclude patients with multiple primaries tumors (n=719)

Exclude patients with incomplete survival data, unknown surgery, unknown chemotherapy， unknown radiation，unknown tumor size，unknown remote metastasis(n=512)

The First Affiliated Hospital of Wenzhou Medical University (n=362)

The Second Affiliated Hospital of Wenzhou Medical University (n=150)
