# Supplementary figures and images for: A prognostic nomogram to predict survival in elderly patients with small-cell lung cancer: a large population-based cohort study and external validation
Source: BMC Cancer. 2022 Dec 6;22:1271. doi: 10.1186/s12885-022-10333-9 (PMC9724365; doi:10.1186/s12885-022-10333-9)

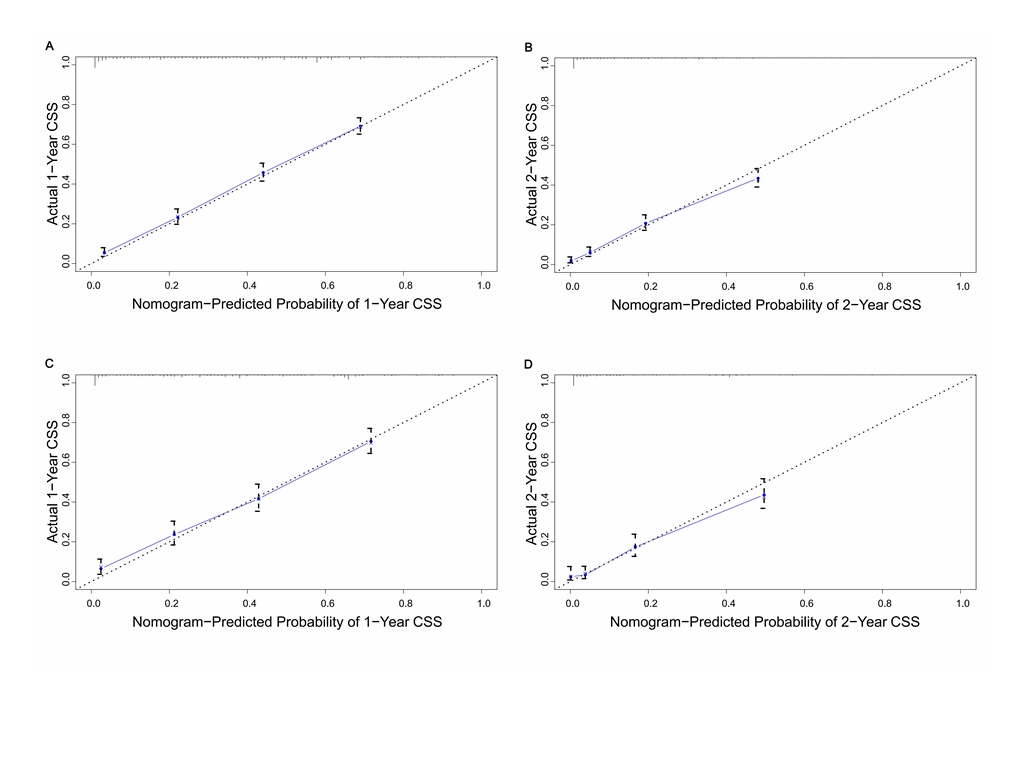

Supplement: Supplementary file 2 — Additional file 2: Supplementary Fig. 1. Calibration plots of the nomogram for 1-, and 2-year CSS prediction of the training cohort (A–B) and internal validation cohort (C–D). [file 12885_2022_10333_MOESM2_ESM.jpg]

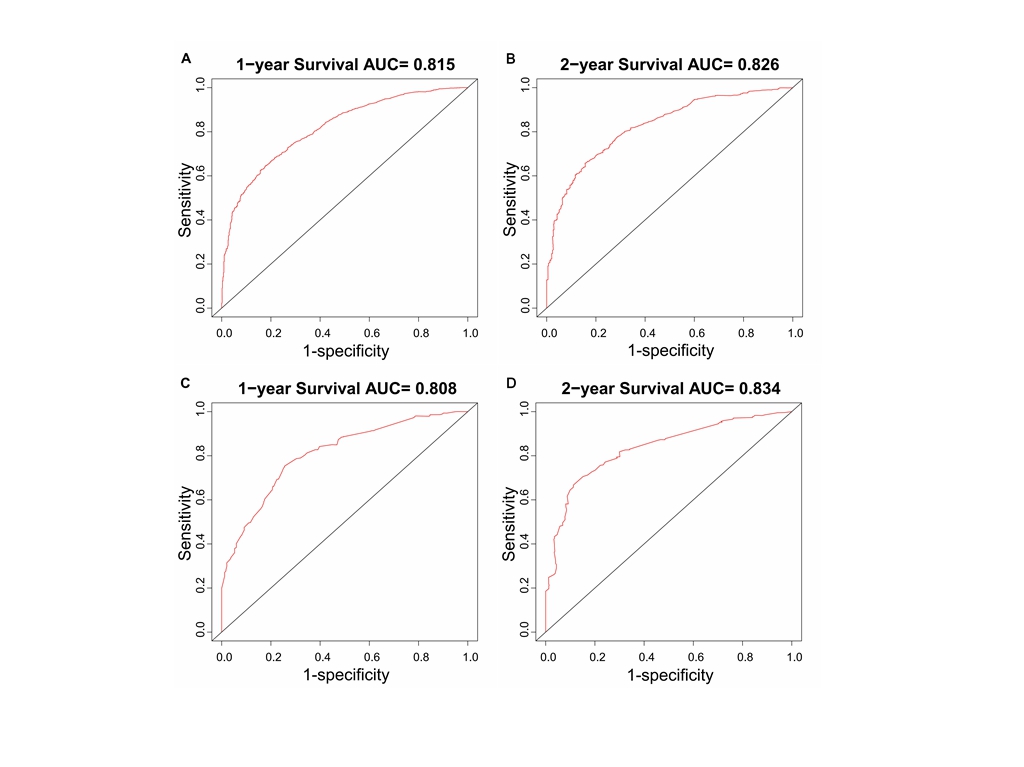

Supplement: Supplementary file 3 — Additional file 3: Supplementary Fig. 2. The ROC curves of the nomograms for 1-, and 2-year CSS prediction of the training cohort (A–B) and internal validation cohort (C–D). [file 12885_2022_10333_MOESM3_ESM.jpg]

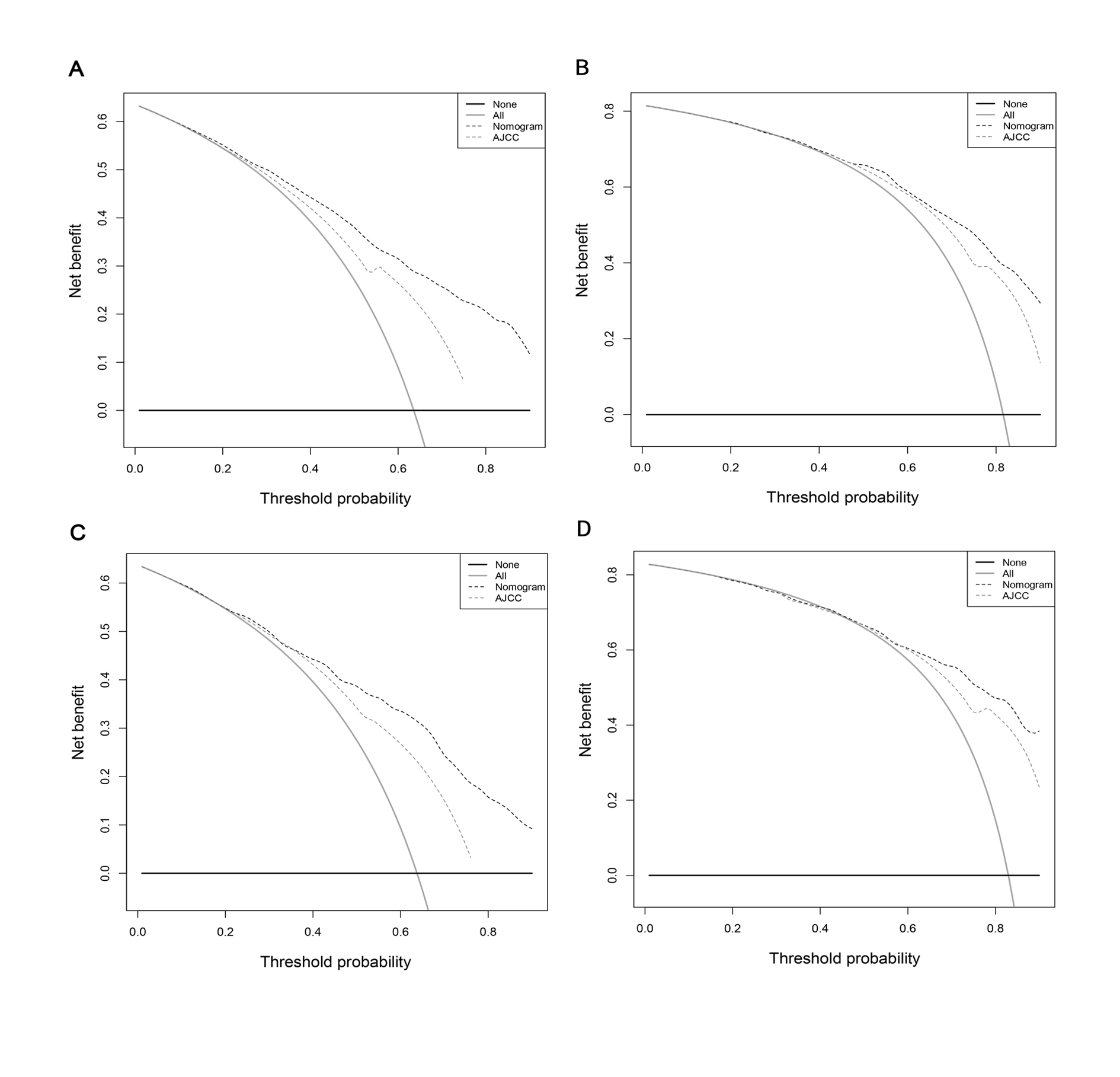

Supplement: Supplementary file 4 — Additional file 4: Supplementary Fig. 3. Decision curve analyses (DCA) of the nomogram and 7th AJCC TNM staging system for 1-year (A, C) and 2-year (B, D) CSS in the training cohort (A–B) and internal validation cohort (C–D). [file 12885_2022_10333_MOESM4_ESM.jpg]

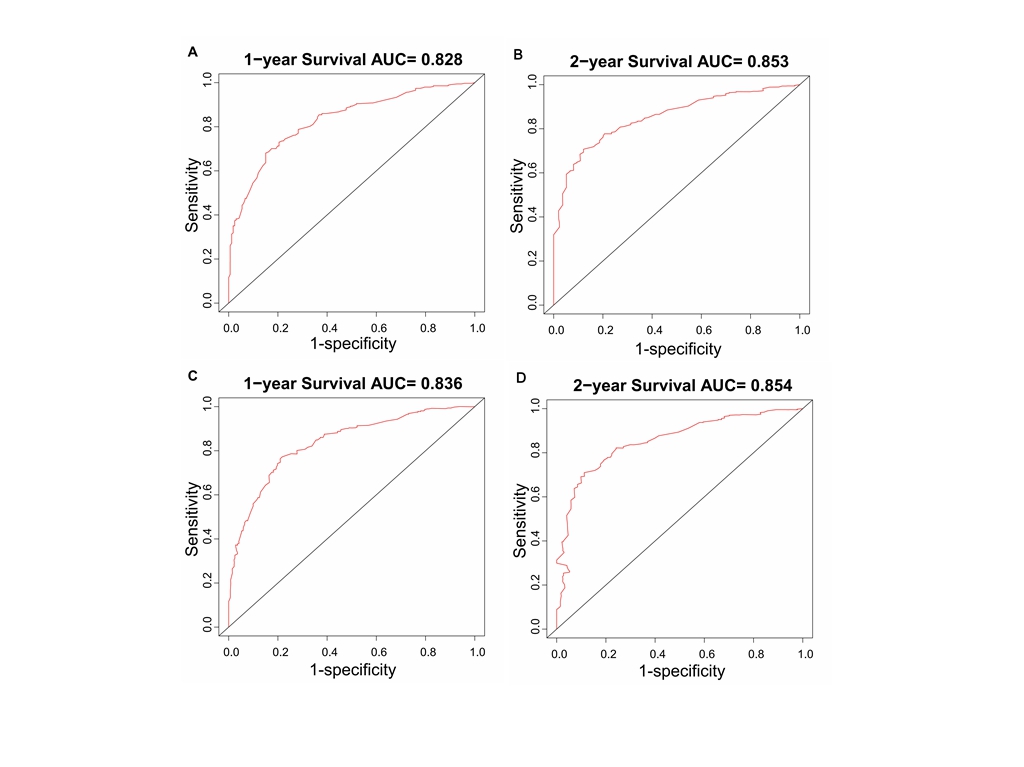

Supplement: Supplementary file 5 — Additional file 5: Supplementary Fig. 4. The ROC curves of the nomograms for 1-, and 2-year OS (A–B) and CSS (C–D) prediction in external validation cohort. [file 12885_2022_10333_MOESM5_ESM.jpg]

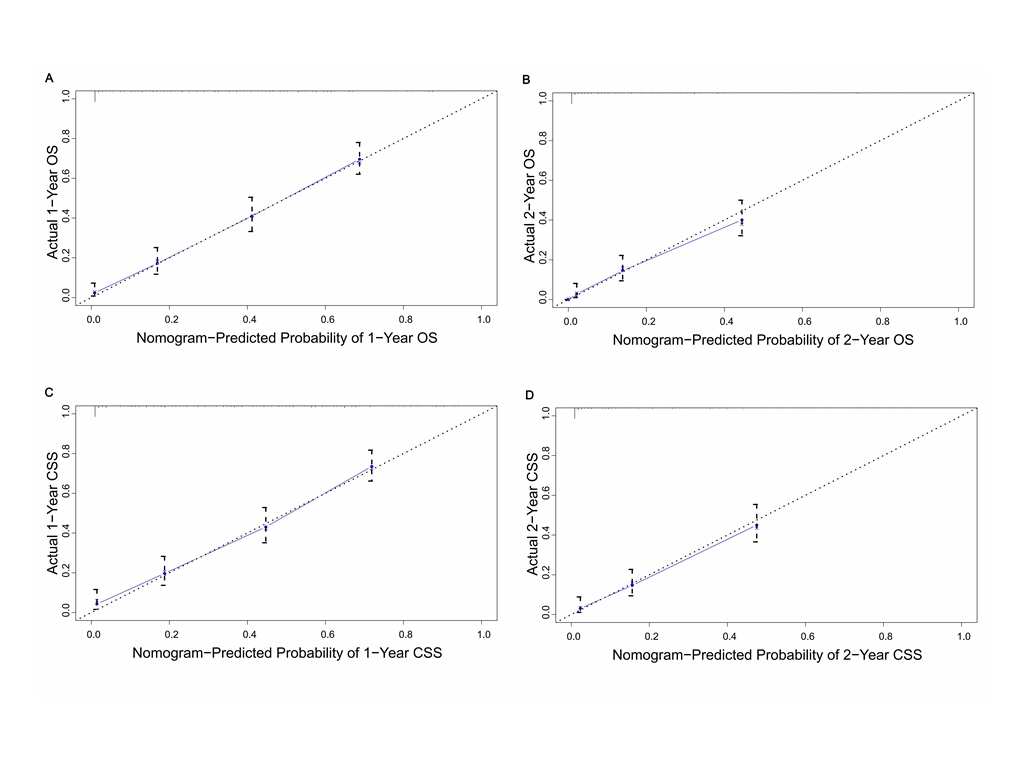

Supplement: Supplementary file 6 — Additional file 6: Supplementary Fig. 5. Calibration plots of the nomogram for 1-, and 2-year OS (A–B) and CSS (C–D) prediction in external validation cohort. [file 12885_2022_10333_MOESM6_ESM.jpg]
