# Supplementary material for: A prognostic nomogram to predict survival in elderly patients with small-cell lung cancer: a large population-based cohort study and external validation
Source: BMC Cancer. 2022 Dec 6;22:1271. doi: 10.1186/s12885-022-10333-9 (PMC9724365; doi:10.1186/s12885-022-10333-9)
Supplement: Supplementary file 7 — Additional file 7: Supplementary Table 1. Patient characteristics of external validation cohort. [file 12885_2022_10333_MOESM7_ESM.docx]

**Supplementary table 1. Patient characteristics of external validation cohort**

| **Variables** | **External validation（n=512）** |
| --- | --- |
| Age（year）n(%) |  |
| 60-69 | 245(47.9) |
| 70-79 | 210(41.0) |
| ≥80 | 57(11.1) |
| Sex n(%) |  |
| Female | 275(53.7) |
| Male | 237(46.3) |
| AJCC TNM stage(7th) n(%) |  |
| I | 25(4.9) |
| II | 43(8.4) |
| III | 146 (28.5) |
| IV | 298(58.2) |
| Surgery n(%) |  |
| No | 460(89.9) |
| Yes | 52(10.1) |
| Chemotherapy n(%) |  |
| No | 103(20.1) |
| Yes | 409 (79.9) |
| Radiation n(%) |  |
| No | 267(44.0) |
| Yes | 287(56.0) |
| Bone metastasis n(%) |  |
| No | 438(85.6) |
| Yes | 74(14.4) |
| Brain metastasis n(%) |  |
| No | 376(73.4) |
| Yes | 136(26.6) |
| Liver metastasis n(%) |  |
| No | 376(70.1) |
| Yes | 153(29.9) |
| Size n(%) |  |
| ≤3cm | 155(30.3) |
| 3.1-5cm | 159(31.0) |
| 5.1-7cm | 97(21.7) |
| ＞7cm | 87(17.0) |

AJCC, American Joint Committee for Cancer; TNM, Tumor-Node-Metastasis.
